# Automatic Bone Removal in CBCT Scans of the Body Trunk: Thorax, Abdomen, and Pelvis

**DOI:** 10.1007/s00270-025-04293-3

**Published:** 2026-01-06

**Authors:** Hinrich Winther, Sabine Maschke, Lena Becker, Cornelia Dewald, Marcel Eicke, Tobias Jakobs, Roman Kloeckner, Axel Schmid, Frank Wacker, Bernhard Meyer

**Affiliations:** 1 Department of Diagnostic and Interventional Radiology, Hannover Medical School, Carl-Neuberg-Straße 1, 30659 Hannover, Germany; 2 Clinic of Interventional Radiology, Hospital Barmherzige Brueder, 93049 Munich, Germany; 3 Institute of Interventional Radiology, University Hospital Schleswig-Holstein Campus Lübeck, Lübeck, Germany; 4 Institute of Radiology, University Hospital Erlangen, Friedrich-Alexander University Erlangen-Nuremberg (FAU), Erlangen, Germany

**Keywords:** Cone beam computed tomography, CBCT, Bone removal, Image guidance, Vessel visualization, Image segmentation, Deep learning

## Abstract

**Purpose:**

To evaluate a fully automated bone removal software for cone beam computed tomography (CBCT) of the thorax, abdomen, and pelvis, enhancing vascular visualization by eliminating bone interference and improving diagnostic quality.

**Material and Methods:**

1035 CBCT scans from adults age 66.5 ± 11.9 18–87 years (mean ± std min–max) across nine centers were retrospectively analyzed, divided into training (*n* = 855, 515 abdomen, 229 pelvis, 111 thorax) and testing (*n* = 180, 60 for each region, 114 male, 53 female, 13 unknown). Manual bone segmentation was performed using ITK-SNAP. A modified 3D U-Net was trained and clinically evaluated through multireader analysis using ordinal scales from 1 (perfect) to 4 (not usable) bone subtraction (B-rating) and erosion of non-target structures (V-rating) in addition to a vessel assessment (VA-rating), categorizing the subtracted image as “better” (1), “same” (2), or “worse” (3). Quantitative metrics include Sørensen–Dice coefficient and intersection over union (IoU).

**Results:**

The software demonstrated high accuracy with a B-rating of 1.01 ± 0.07 and a V-rating of 1.02 ± 0.13, indicating minimal erosion of non-target structures. A VA-rating of 1.0 ± 0 suggests an improved vessel assessment and the depiction of contrast material deposition, enhancing the diagnostic quality of CBCT images. Quantitative analysis closely matched the manual expert delineation (Sørensen–Dice coefficient 0.95 ± 0.02, IoU of 0.9 ± 0.03).

**Conclusion:**

The software provides robust, fully automated bone removal in CBCT scans. This technology may enhance vascular system visualization without compromising non-target structures, potentially improving the accuracy and efficiency of interventional and diagnostic radiology procedures.

**Graphical Abstract:**

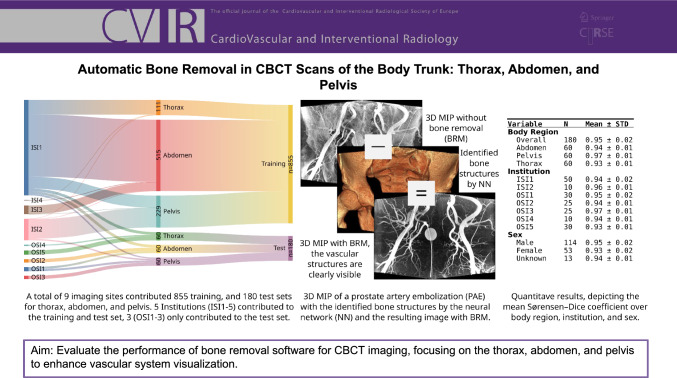

**Supplementary Information:**

The online version contains supplementary material available at 10.1007/s00270-025-04293-3.

## Introduction

Bone removal (BRM) techniques play an important role in CT angiography (CTA) of the head [1], body trunk [2], and the lower extremities [3], as well as in cone beam computer tomographies (CBCT) of the head and trunk. BRM enables the rapid evaluation of the vascular system and is often used to produce overview reconstructions of the scan, utilizing maximum intensity projections (MIP) or volume rendering techniques (VRT). Without BRM, the resulting visualization of the vasculature is often obscured by overlapping bone structures, resulting in severely impaired and therefore inadequate images, impacting both the diagnostic work-up and the peri-procedural guidance [4].

The possible uses for BRM in CBCT scans of the body trunk are manifold and include both diagnostic and therapeutic interventions, e.g., bleeding control, prostate embolization (PAE), pulmonary balloon angioplasty (BPA), and different liver interventions [5–7]. Additionally, it has been suggested that, for transarterial chemoembolization (TACE), the interventional radiologist may catheterize tumor-feeding arteries with CBCT guiding technology more readily than without [8–10]. In the case of BPA, CBCT guidance, using VRT-based road maps, enables a safe procedure with a low risk of severe complications [11].

Different approaches exist to create a bone-free 3D-rendered image: Using thresholding, software-based methods can be employed on post-processing datasets to automatically separate bones concealing relevant information in the field of view (FOV). However, as these technologies rely on specific attenuation values to distinguish between bone and soft tissue, inaccuracies can lead to inadequate removal of bone or incorrect removal of artery segments [12].

CBCT combines DSA and CTA imaging features, but with a higher spatial resolution at the cost of a higher image noise [13]. BRM is particularly important and challenging because it allows arbitrary rotation of the 3D CBCT dataset, offering a luminal view of the vessel. It also displays information about the perivascular soft tissue, helping realize this imaging modality’s potential. Especially in complex procedures, imaging the target structure in as much detail as possible is crucial. However, manual segmentation is usually necessary to achieve BRM in CBCT images of the body trunk, rendering time-consuming post-processing mandatory, requiring two to five minutes [11, 14]. Few prior efforts have been published to achieve automated BRM without relying on the differentiation of attenuation values, e.g., by Wang et al., who have demonstrated the applicability of a deformable model to identify the body cavity on dual-phase CBCT to subtract the rib cage and spine for transarterial chemoembolization (TACE) of the liver [15]. However, to our knowledge, a widely applicable solution for BRM for CBCT of the body trunk is not available.

This study aims to evaluate the performance of a newly developed bone removal software for CBCT imaging, focusing on the thorax, abdomen, and pelvis to enhance vascular system visualization by eliminating bone structure interference, thereby improving diagnostic quality.

## Material and Methods

### Study Cohort

1035 CBCT scans, each from an individual subject, from the thorax, abdomen, and pelvis of adults (age > 18 years), were retrospectively included in the study cohort from 9 different centers. Each center has been asked to provide chronologically consecutive exams to get a representative real-world sample. The angiography system used in all centers in this study was the Axiom Artis (Siemens Healthcare) with the HU normal reconstruction kernel, except for two exams where the HU sharp reconstruction kernel was used. Non-diagnostic scans due to heavy motion artifacts were excluded. The data have been split into a test and a training set chronologically on a per-center basis, with the oldest data (from 05/2013) being used for training and the newest for testing (until 03/21). Four centers ISI1-4 (**I**n **S**ample **I**nstitution, the center’s data are included in both training and testing) contributed training (*n* = 855) and testing (*n* = 60); five centers OSI1-5 (**O**ut of **S**ample **I**nstitution, the center provides testing data only) contributed only testing data (*n* = 120). The five OSI centers were deliberately not included in the training to estimate any present overfitting of the model, which center was included in the training and testing, as well as in testing only, was decided based on the availability of scans regarding the body region, as many centers strongly focus on specific exam types and the resulting body region. All test cases except three exams of ISI1 have been performed with a contrast agent; however, 21 additional cases show a parenchymal contrast. A detailed breakdown of the study cohort is provided in Table 1.

**Table 1:** Patient cohort characteristics

Split	Institution	Age (mean ± std min–max)	Sex (m/f/o)	#Samples	#ABD	#PEL	#THO	kVp (mean ± std min–max)	FOV mm^2^ (mean ± std min–max)
Train	ISI1	62.6 ± 14.7 (18–93)	414/226/0	640	448	86	106		
	ISI2	70.1 ± 10.0 (47–93)	144/1/0	145	2	141	2		
	ISI3	68.8 ± 9.5 (51–84)	54/9/0	63	62	1	0		
	ISI4	nan	0/0/7	7	3	1	3		
	Overall	64.4 ± 14.0 (18–93)	612/236/7	855	515	229	111		
Test	ISI1	65.1 ± 14.0 (29–84)	27/23/0	50	20	10	20	94.3 ± 5.4 (90–108)	249.2 ± 2.3 (240–252)
	ISI2	71.6 ± 8.1 (54–80)	10/0/0	10	0	10	0	106.5 ± 3.2 (101–111)	203.8 ± 17.0 (198–252)
	OSI1	65.0 ± 11.1 (33–84)	21/9/0	30	15	15	0	106.6 ± 8.1 (90–124)	251.2 ± 0.2 (250–251)
	OSI2	69.5 ± 11.8 (51–87)	8/4/13	25	25	0	0	104.1 ± 8.2 (90–119)	251.1 ± 0.0 (251–251)
	OSI3	68.0 ± 13.1 (18–87)	25/0/0	25	0	25	0	105.6 ± 6.4 (95–114)	246.5 ± 19.5 (198–271)
	OSI4	68.5 ± 5.3 (62–77)	10/0/0	10	0	0	10	90.0 ± 0.0 (90–90)	240.3 ± 0.6 (240–241)
	OSI5	65.2 ± 10.7 (42–81)	13/17/0	30	0	0	30	108.4 ± 3.5 (90–109)	251.4 ± 2.8 (236–252)
	Overall	66.5 ± 11.9 (18–87)	114/53/13	180	60	60	60	102.1 ± 8.6 (90–124)	246.8 ± 13.5 (198–271)

The institution prefix denotes if the center contributed to the training and test set (ISI) or to the test set only (OSI). Sex is given in male (m), female (f), and anonymized (o). The main body region is given in thorax (THO), abdomen (ABD), and pelvis (PEL). Not a number (nan) is given when the anonymization process discarded the relevant information

Each center anonymized the image studies during the export before inclusion in the dataset. Finally, the data were split into 855 training and 180 test cases.

### Data Annotation

We used ITK-Snap [16] version 3.8 to manually segment the bony structures of all 1035 CBCT scans. In ITK-SNAP, this was done using tools such as the “Paintbrush” tool, which allowed us to manually delineate the boundaries of the bone structure on each slice. This process is meticulous and time-consuming, requiring precision to accurately delineate the bones’ anatomical structure. The size of the paintbrush can be adjusted for finer control over the segmentation, especially in areas where bone structures are in proximity or intersect with other anatomical structures, such as vessels. Throughout this process, it was crucial to maintain consistency in segmenting the bone structures across all slices to create a cohesive and accurate 3D representation of the structure. We tried to control this process as closely as possible by employing technical solutions, performing structural component analysis, and including guidelines to review the segmentation in a multiplanar reconstruction and 3D VRT.

### Machine Learning

The architecture implemented in PyTorch version 1.8.1 is based on the 3D U-Net model as proposed by Çiçek et al. [17, 18], with an input layer dimensionality increased to 256 × 256 × 256 neurons. The architecture utilizes a symmetrical design that comprises both downsample and upsample blocks. Each downsample block features a batch normalization process followed by two sets of 3 × 3 × 3 convolutions, employing a stride of 3 with zero padding. Upsample blocks incorporate a resampling layer that utilizes linear interpolation to match the resolution of corresponding downsample blocks, succeeded by two 3 × 3 × 3 convolutions with zero padding. Following the recommendations of He et al. [19], traditional ReLU activation functions were replaced with PReLU across the network to enhance model learning dynamics.

The model was trained using weak binary cross-entropy as the objective function. Training commenced with a learning rate of 1*e*^−3^, which was gradually decreased to 1*e*^−6^. Each training epoch comprised 200 virtual samples, generated dynamically through image augmentation techniques, including linear (rotation, shearing, resizing) and nonlinear transformations (local displacement), with a batch size of eight. Optimization was facilitated through the adaptive moment estimation (Adam) method [20].

The training infrastructure consisted of a DGX1 system equipped with eight NVIDIA V100 GPUs, each boasting 32 GB of video memory. The training duration extended over a period of seven days.

Upon completion of the training phase, the modified 3D U-Net model was applied to a set of 180 test cases for image segmentation. Notably, the process omitted conventional pre-processing steps such as denoising or anatomical extraction to assess the raw segmentation capability of the model directly.

### Qualitative Evaluation

**Fig. 1:**
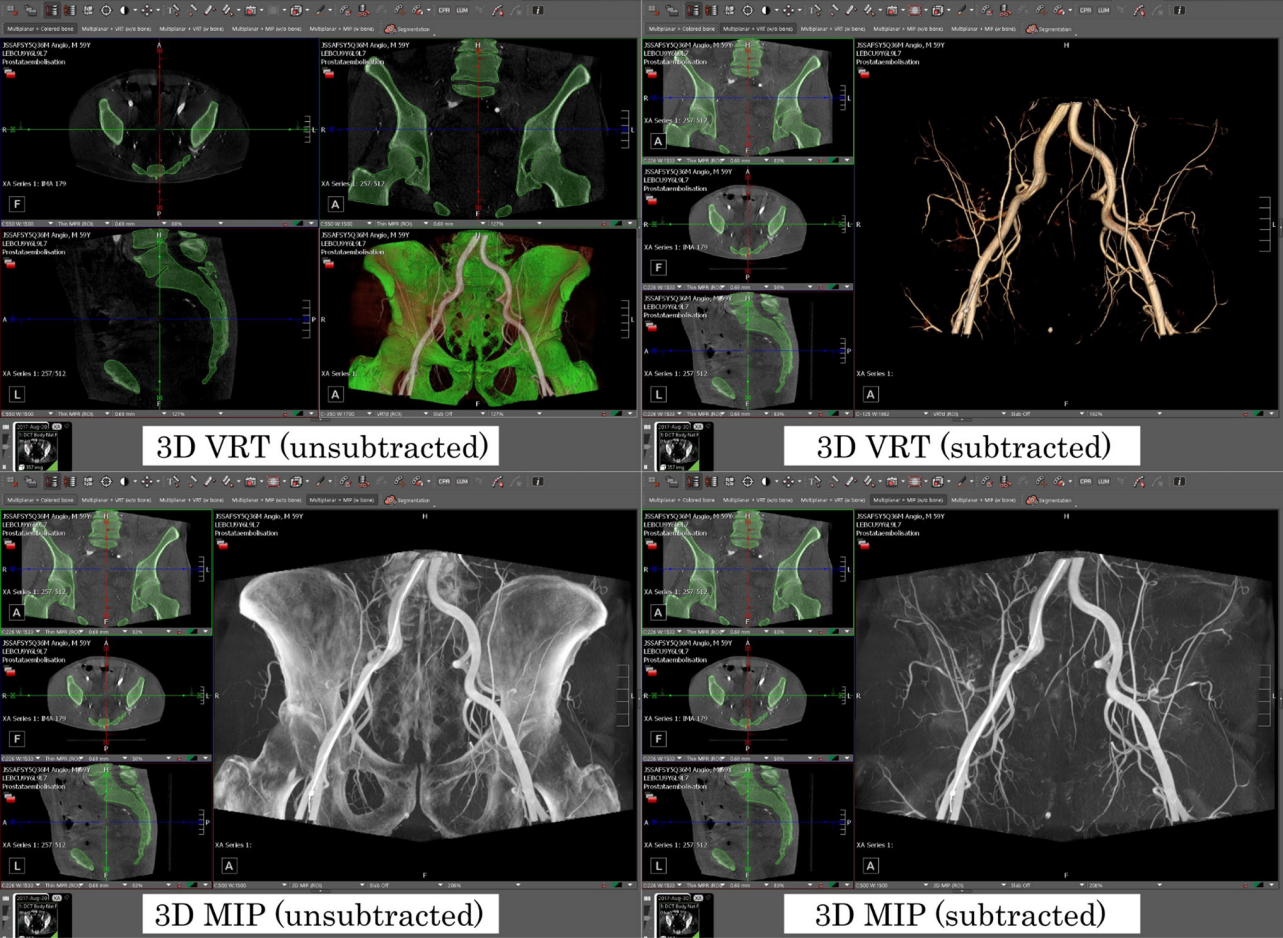
Example test case, depicting the different views for the reader: 3D maximum intensity projection (MIP) and volume rendering technique (VRT) unsubtracted and subtracted

We performed a qualitative multireader analysis, in which three radiological experts (board-certified radiologists with 15, 10, and 8 years of experience in IR) visually evaluated the clinical applicability of the fully automated bone segmentation for bone subtraction. The 180 test cases (see Table 1), 60 each for thorax, abdomen, and pelvis, were pre-loaded into a DICOM viewer (Visage Imaging Inc., Visage 7, Build 3416). A standardized hanging protocol was developed, allowing the reader to quickly switch between the subtracted and the unsubtracted view, featuring multiplane reconstruction (MPR), maximum intensity projection (MIP), and volume rendering technique (VRT) visualization, as depicted in Fig. 1. The quality of the bone subtraction was evaluated using an ordinal scale in two dimensions: completeness of the bone subtraction (B-rating) and erosion of non-target structures, such as soft tissue (V-rating). The criteria for the B- and V-ratings are depicted in Table 2.

**Table 2:** Definition of the evaluation criteria for the manual reading of the test cases

B-Rating	Definition
B1	*Complete bone mask* In MIP and VRT, no residuals of the bone are seen In MPR, at most, small subtraction residues close to the bone can be detected—without a significant correlation in MIP or VRT Soft tissue visualization using VRT and/or MIP is possible without obscuring bony structures
B2	*Irrelevant residual bone, no need for manual post-processing* In MIP and VRT, only irrelevant residuals (due to location or size) of the bone are seen Soft tissue visualization using VRT and/or MIP is possible without relevant obscuring bony structures
B3	*Relevant residual bone, manual post-processing necessary* In MIP and VRT, relevant residuals of the bone are seen obscuring major, but not all soft tissue information Soft tissue visualization using VRT and MIP is obscured in major parts by bony structures so that manual cropping at the workstation would be (deemed) necessary The user would have to apply corrective actions: Manual cropping of residual bone
B4	*No (useful) bone mask was calculated* No bone mask was calculated or an unusable bone mask even with manual correction was calculated Soft tissue visualization using VRT and/or MIP is massively obscured by bony structures The user would have to apply corrective actions: The bone mask cannot be used and has to be reset

V-Rating	Definition
V1	*No overreaching of the bone mask* In MPR, MIP, and VRT, there is no relevant truncation of soft tissue information At most, negligible truncation of soft tissue close to the bone can be detected; e. small arteries running next to or into the bone are affected Soft tissue visualization using VRT and MIP is possible without restrictions
V2	*Mild overreaching, Minor truncation of soft tissue information:* In MPR, MIP, and VRT, there is negligible truncation of the soft tissue: There are only clinically irrelevant truncations or only marginal truncations of relevant vessels Soft tissue visualization using VRT and MIP is provided without relevant restrictions
V3	*Relevant overreaching of the bone mask* In MPR, MIP, and VRT, there is local truncation of clinically relevant information of the soft tissue In case of truncation of clinically relevant vessels, the vessel’s central continuity is affected Soft tissue visualization using VRT and MIP is limited due to truncation of clinically relevant structures The user would have to apply at least one of the following corrective actions: Manual cropping of the bone mask at the workstation would deem necessary In areas of relevant truncation, the original data would have to be used to assess relevant information In areas of relevant truncation, navigation would have to be supplemented (e. g. by graphic representations of the course of the vessels) to perform the clinical procedure
V4	*Massive overreaching of the bone mask* In MIP and VRT, relevant truncation of clinically relevant information makes 3D visualization impossible. The bone mask is insufficient and cannot be used Soft tissue visualization using VRT and MIP is not possible The user would have to apply corrective actions: The bone mask cannot be used and has to be reset

MIP, Maximum intensity projection; VRT, volume rendering technique; MPR, multiplanar reconstruction

Additionally, we asked for a clinical appraisal of whether bone subtraction improves vessel assessment (VA-rating), classifying the result as “better,” “same,” or “worse.” 24 of the test cases were excluded from the VA-rating due to native (*n* = 3) or parenchymal contrast (*n* = 21).

The final score for each case across the three readers was determined by majority voting, yielding a single consensus B-, V-, and VA-rating per case.

### Inter-Rater Agreement

In our dataset, almost all ratings fall in a single category (score = 1) with only a handful of discordant observations (score = 2). Under such extreme class imbalance, Krippendorff’s *α*, which corrects observed disagreement by the disagreement expected from the marginal distributions, suffers from the well-known prevalence/bias paradox. The expected-by-chance disagreement becomes vanishingly small, so even a few mismatches can yield *α* ≈ 0 or slightly negative despite high raw agreement.

Intraclass correlation coefficients (ICC) are likewise ill-suited here. ICC assumes at least interval-level, approximately normal, and homoscedastic measurement. It quantifies consistency in a linear model and is highly sensitive to restricted range (low between-item variance) and scale skew, both present with a bounded 1–4 ordinal scale dominated by a single category. In this setting, ICCs can be unstable and misleading, conflating lack of variability and reliability.

We report percent agreement as a primary descriptive metric to provide an interpretable summary that directly reflects how often readers make the same judgment. Percent agreement (PA) is unaffected by prevalence/bias corrections.

### Quantitative Evaluation

Additionally, we present a quantitative analysis with overlap evaluation metrics, including the Dice coefficient, also referred to as the Sørensen–Dice coefficient, and the intersection over union (IoU), commonly known as the Jaccard index. For these metrics, 0 means no match and 1 means a perfect match.

## Results

### Qualitative Analysis

The qualitative results depicted in Table 3 demonstrate that the model is able to accurately delineate the bone structure with an overall B-rating of 1.01 ± 0.07 (mean ± std) while not eroding relevant non-target structures in a major way with an overall V-rating of 1.02 ± 0.13.

**Table 3:** Summary statistics of the visual multireader analysis

	Overall	Reader 1	Reader 2	Reader 3
*B-rating*				
Mean ± std	1.01 ± 0.07	1.02 ± 0.13	1.04 ± 0.19	1.01 ± 0.07
Median	1	1	1	1
Min/max	1/2	1/2	1/2	1/2
#Test cases	180	180	180	180
#Rating 1	179	177	173	179
#Rating 2	1	3	7	1
*V-rating*				
Mean ± std	1.02 ± 0.14	1.02 ± 0.18	1.09 ± 0.28	1.02 ± 0.15
Median	1	1	1	1
Min/max	1/2	1/3	1/2	1/2
#Test cases	156	156	156	156
#Rating 1	177	177	164	176
#Rating 2	3	2	16	4
#Rating 3	0	1	0	0
*VA-rating*				
Mean ± std	1.00 ± 0.00	1.00 ± 0.00	1.00 ± 0.00	1.00 ± 0.00
Median	1	1	1	1
Min/max	1/1	1/1	1/1	1/1
#Test cases	156	156	156	156

The B-rating evaluates the completeness of the bone mask, the V-rating evaluates if there is an overreaching of the bone mask, eroding relevant structures, such as vessels. The vessel assessment (VA) rating is a clinical appraisal of whether bone subtraction using BRM improves vessel assessment. The overall scores have been calculated by performing a majority voting of reader 1, 2, and 3. The number of test cases for the B-rating (# 180) differs from the V- and VA-rating (156) because no or no arterial contrast agent was applied in 24 cases

**Fig. 2:**
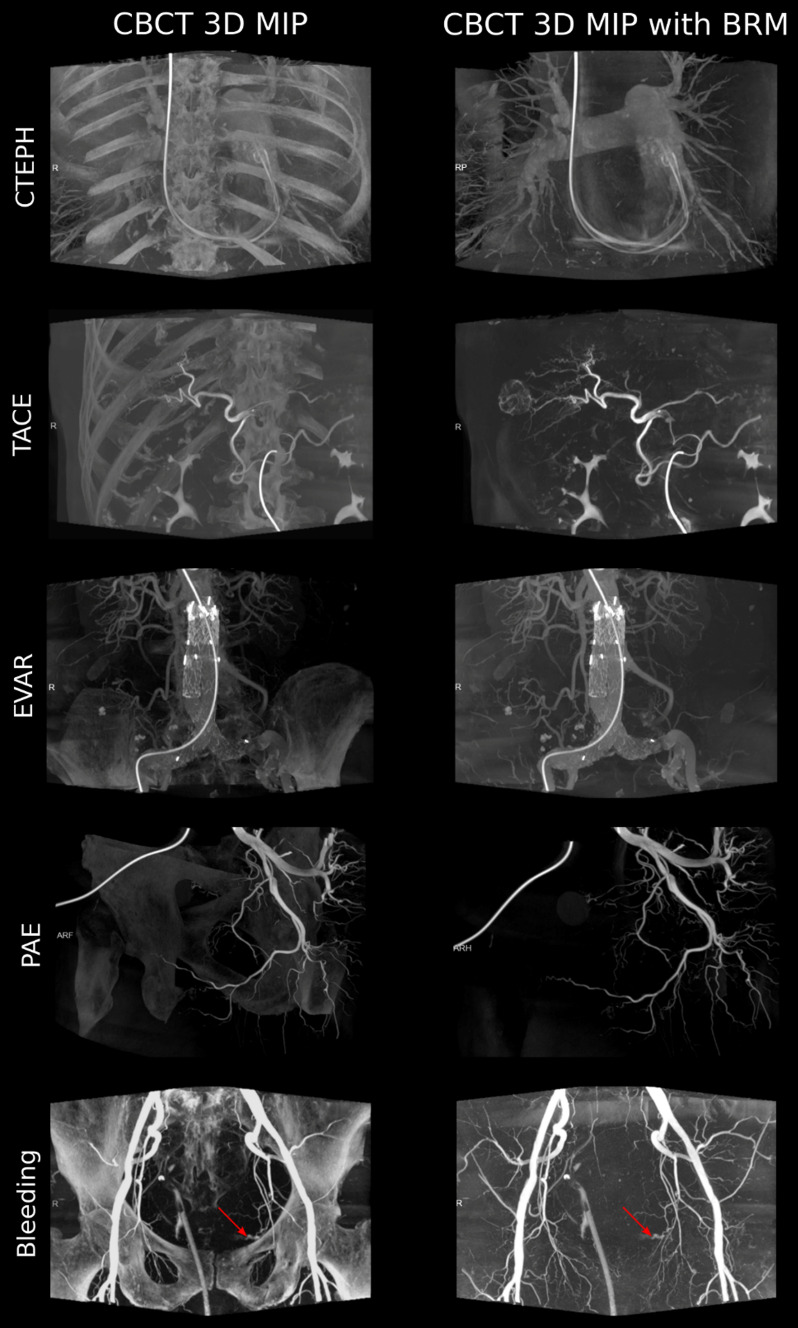
The study compares 3D maximum intensity projections (MIP) of cone beam CT (CBCT) scans, both with and without bone removal (BRM). These scans were performed for various medical procedures. One scan of the thorax was conducted to treat chronic thromboembolic pulmonary hypertension (CTEPH). Another scan of the upper abdomen was used for transarterial chemoembolization (TACE). A scan of the lower abdomen was carried out for endovascular aneurysm repair (EVAR). Additionally, scans of the pelvis were performed for prostate artery embolization (PAE) and for the detection of bleeding. The red arrows point to the bleeding

A selection of examples from the test set for the bone removal software for the thorax, abdomen, and pelvis is depicted in Fig. 2.

### Inter-Rater Agreement

The PA for all three readers, where all the readers agree on the score per observation, is ≈ 94.4% for the B-rating, ≈ 89.1% for the V-rating, and 100% for the VA-rating. A detailed per-reader pair analysis is provided in Table 4.

**Table 4:** Detailed inter-rater analysis with the percent agreement (PA) given for each rater combination for the B-rating, V-rating, and VA-rating

Reader A	Reader B	Reader C	PA (B-rating) [%]	PA (V-rating) [%]	PA (VA-rating) [%]
Reader 1	Reader 2		94.44	89.10	100
Reader 1	Reader 3		97.78	97.44	100
Reader 2	Reader 3		96.67	91.03	100
Reader 1	Reader 2	Reader 3	94.44	89.10	100

### Quantitative Analysis

We used the manual segmentation of the 180 CBCT test cases for quantitative analysis. The results depicted in the electronic supplementary material (ESM). Table 1 demonstrates a good alignment of the fully automated bone segmentation using the trained model, with the manual delineation of the bone structure, with a Sørensen–Dice coefficient of 0.95 ± 0.02 (mean ± std) and an IoU of 0.9 ± 0.03 overall. The ESM Table 1 lists a detailed subgroup analysis based on body region, institution, and sex.

## Discussion

This study presents a promising advancement in applying fully automated bone removal software for CBCT angiography imaging. The development and deployment of this software offer a potential solution to the longstanding challenge of bony interference in imaging, which has traditionally compromised the clarity and diagnostic utility of such acquisitions, especially in areas densely populated by bone structures like the thorax, abdomen, and pelvis.

The qualitative and quantitative results obtained from the evaluation of 180 test cases indicate that the software achieves high accuracy in bone subtraction and maintains the integrity of non-target structures, such as soft tissues. The three readers concluded that all test cases benefited from applying the bone removal with a VA-rating of 1.0 ± 0.0 (mean ± std). This is also reflected in a high Sørensen–Dice coefficient of 0.95 ± 0.02. Additionally, bone subtraction improved vessel assessment and depiction of contrast material deposition in CBCT.

CBCT guidance has been shown to be beneficial in a wide range of angiographic interventions: Based on CBCT images, the contrasted vessels, e.g., pulmonary arteries in BPA, pelvic arteries in prostatic artery embolization, or hepatic arteries in TACE, can be visualized as a three-dimensional vascular tree by a VRT [5, 11, 21, 22]. This can be used as a graphic overlay with semitranslucent opacity and blended with live fluoroscopic images, allowing the use of CBCT vessel information for a real-time peri-interventional roadmap [5]. The value of CBCT for the pre- and peri-procedural management of super-selective conventional TACE has been highlighted recently in a recommendation from a panel of international expert physicians [21]. Furthermore, CBCT can also play a crucial role in pre-procedural planning and peri-procedural guidance during BPA, providing high-resolution imaging of the entire pulmonary vasculature [23]. Regarding PAE, pre-interventional CBCT can help identify the prostatic arteries, which, due to adjacent arterial branches arising from the internal iliac artery and variable origin of the prostatic artery, can be challenging [22].

However, as bones may obstruct the visualization of the vasculature and, thus, limit the expressiveness of the examination, BRM is usually mandatory. Currently, BRM requires manual interaction, potentially causing an inefficient workflow and delaying the procedure.

Several approaches have been made to automate bone segmentation. The vast majority used gray-value-based thresholding [24, 25], which regularly removes contrast-enhanced vessels, especially in proximity to bone structures. This effect is more pronounced with CBCT, which has higher image noise than CTA. This reduces the general usability of automated BRM for CBCT based on threshold values.

Our fully automated bone removal software, based on semantic segmentation, can facilitate BRM for the whole body trunk, thus offering improvements for manifold interventions. This allows a precise differentiation between vessel and bone, even in the peripheral sections of the vascular tree, regardless of the higher image noise compared to CTA. Additionally, the software may be integrated into existing image processing systems and provides real-time results.

There are study limitations inherent in the field of automated image segmentation. The reliance on specific attenuation values to distinguish between bone and soft tissue can introduce inaccuracies, highlighting the need to refine algorithms and segmentation techniques continuously. Furthermore, the study’s focus on a diverse study cohort from multiple centers adds to the robustness of the findings. However, it also suggests potential variability in scan quality and segmentation challenges across different settings.

Future research should address these limitations by exploring more sophisticated algorithms that can adapt to the variability in imaging data and further reduce the potential for incorrect removal of critical structures. Additionally, expanding the application of this technology to other imaging modalities and anatomical regions could broaden its utility in medical imaging. Integrating such automated BRM software into clinical practice could significantly enhance the efficiency and accuracy of diagnostic processes, particularly in the rapid evaluation of emergency conditions where time is of the essence.

## Conclusion

This study demonstrates the feasibility and effectiveness of fully automated bone removal software in improving the clarity and diagnostic utility of CBCT images. By reducing the interference of bone structures without compromising the visibility of essential soft tissues and vascular systems, this technology represents a valuable tool in the interventional and diagnostic imaging arsenal.

## Supplementary Information

Below is the link to the electronic supplementary material.

Supplementary file1 (DOCX 124 KB)
