# Supplementary material for: Automatic Bone Removal in CBCT Scans of the Body Trunk: Thorax, Abdomen, and Pelvis
Source: Cardiovasc Intervent Radiol. 2026 Jan 6;49(3):636–45. doi: 10.1007/s00270-025-04293-3 (PMC12963259; doi:10.1007/s00270-025-04293-3)

**ESM Table 1:** Overlap and subgroup statistics, including Dice coefficient, also referred to as the Sørensen–Dice coefficient, and the Intersection over Union (IoU), also commonly known as the Jaccard index. Additionally, the B-, V- and VA-ratings for each subgroup are given.


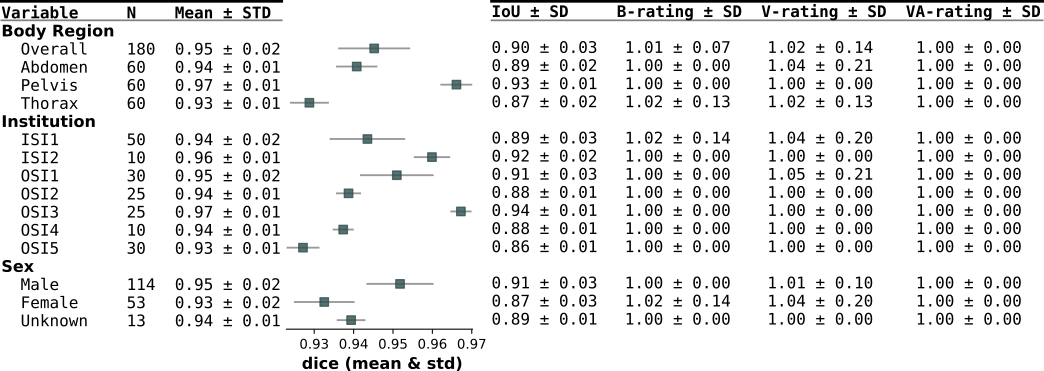

Supplement: Supplementary file 1 — Supplementary file1 (DOCX 124 KB) [file 270_2025_4293_MOESM1_ESM.docx]
